# Bushen huoxue decoction inhibits RANKL-stimulated osteoclastogenesis and glucocorticoid-induced bone loss by modulating the NF-κB, ERK, and JNK signaling pathways

**DOI:** 10.3389/fphar.2022.1007839

**Published:** 2022-11-18

**Authors:** Yamei Liu, Binlan Fu, Xiaoman Li, Chen Chen, Xican Li, Liangliang Xu, Bin Wang

**Affiliations:** ^1^ School of Basic Medical Science, Guangzhou University of Chinese Medicine, Guangzhou, China; ^2^ Laboratory of Orthopedics and Traumatology, Lingnan Medical Research Center, Guangzhou University of Chinese Medicine, Guangzhou, China; ^3^ School of Chinese Herbal Medicine, Guangzhou University of Chinese Medicine, Guangzhou, China; ^4^ The First Affiliated Hospital of Guangzhou University of Chinese Medicine, Guangzhou, China; ^5^ Department of Traumatology, The Third Affiliated Hospital of Guangzhou University of Chinese Medicine, Guangzhou, China

**Keywords:** bushen huoxue decoction, osteoporosis, NF-κB pathway, ERK pathway, JNK pathway

## Abstract

Glucocorticoid-induced osteoporosis (GIOP) is the most common form of secondary osteoporosis, which is caused by a disorder in bone metabolism due to excessive activation of osteoclasts. Bushen Huoxue decoction (BHD) is an herbal formula with multiple pharmacological effects, including anti-inflammatory, antioxidant activity and stem cell migration promotion. However, the effect of BHD on osteoclastogenesis has not been reported. In this study, we aimed to elucidate the effect of BHD on RANKL-stimulated osteoclastogenesis and explored its underlying mechanisms of action *in vitro*. Our results show that BHD had no effect on BMMs and RAW264.7 cells viability, but inhibited RANKL-induced osteoclast formation *in vitro*. Furthermore, BHD attenuated RANKL-induced NF-κB, ERK, and JNK signaling. The attenuation of NF-κB, ERK, and JNK activation were enough to impede downstream expression of c-fos and NFATc1 and related specific genes. Meanwhile, we investigated the therapeutic effect of BHD on glucocorticoid-induced osteoporosis (GIOP) mice. The result indicated that BHD prevents glucocorticoid-induced osteoporosis and preserves bone volume by repressing osteoclast activity. Collectively, BHD shows significant osteoclast inhibition and holds great promise in the treatment of osteoporosis.

## Introduction

Glucocorticoids (GCs) are widely used in the treatment of immune and inflammatory diseases, and approximately 0.5–2% of the population are reported to be receiving long-term glucocorticoid therapy (Overman et al., 2013; Silverman et al., 2015; Dineen et al., 2019; Suehs et al., 2021). However, long-term or high-dose use of GCs can lead to various adverse reactions such as diabetes, osteoporosis, myopathy, and others. Among them, glucocorticoid-induced osteoporosis (GIOP) is the most common secondary osteoporosis.

Endogenous GCs regulate key processes at physiological concentrations, including calcium homeostasis in the gut and kidney, skeletal development etc. (Seibel et al., 2013). GCs have been reported to modulate bone biology through a range of distinct mechanisms, and the negative decoupling between bone formation and bone resorption is thought to be critical for early and rapid bone loss (Weinstein et al., 1998; Hofbauer and Rauner 2009; Den Uyl et al., 2011). During the initial phase of GCs treatment, the number and activity of osteoclasts were increased by upregulating the expression of macrophage-colony stimulating factor (M-CSF) and RANKL, while decreasing osteoprotegerin (OPG) mRNA transcription (Hofbauer et al., 1999; Swanson et al., 2006). This process and other effects on IL-6 (Dovio et al., 2006) and interferon-β66 (Takuma et al., 2003) expression may account for the transient increase in bone resorption during the initial phase of GCs treatment. In addition, GCs also exert direct effects on bone formation, such as GCs upregulate the expression of peroxisome proliferator-activated receptor γ2 (PPARγ2) (Ito et al., 2007), Kruppel-like factor 15 (KLF15) (Yang et al., 2018) CCAAT/enhancer binding protein-α (C/EBPα), adipocyte protein 2 (aP2) (Wu, Bucher, and Farmer 1996). The mechanism favors the differentiation of pluripotent precursor cells towards adipocytes in preference to osteoblasts, thus decreasing the number of osteoblasts. Besides, GCs increase the expression of Sclerostin and Dickkopf-l, while suppressing the expression of WNT16 (Ohnaka et al., 2005; Sato et al., 2016; Colditz et al., 2019; Hildebrandt et al., 2020), which leads to a reduction in osteoblastogenesis and bone loss.

Bushen Huoxue decoction (BHD) is a traditional Chinese medicinal formula that was first recorded by Zhao Lian of the Qing Dynasty. It has been reported that BHD has anti-inflammatory and antioxidant effects for the treatment of cardiovascular disease, ovarian and craniocerebral diseases (Ye et al., 2014; Li et al., 2016; Huang et al., 2017; Liu et al., 2019; Feng et al., 2020). What’s more, BHD is widely used in orthopedic conditions such as osteoporosis and osteoarthritis, as it improves blood circulation and relieves swelling in the injured area. Previous studies have shown that BHD relieves osteoporosis primarily in a β-catenin-dependent manner by promoting osteogenic differentiation of growth plate chondrocytes (Xia et al., 2020). BHD can promote the migration of BMSCs and increase osteoblast proliferation, differentiation and mineralization (Shen et al., 2018). The active ingredients of BHD have protective effects on cartilage matrix degradation and inhibit DMM-induced chondrocyte pyrolysis and apoptosis by inhibiting NF-κB signaling (Yu et al., 2021). In addition, BHD has also been reported to inhibit intervertebral disc degeneration (IDD) through MAPK signaling pathway (Feng et al., 2021).

Although previous studies have reported that BHD can treat bone-related disorders by affecting NF-κB as well as MAPK signaling pathways. However, the effects of BHD on the formation and differentiation of osteoclasts and the mechanism of action on GIOP are not well understood. Based on the important effects of NF-κB and MAPK on the formation and differentiation of osteoclasts, we hypothesized that BHD might inhibit the activity of osteoclasts, thereby preventing the occurrence of GIOP. In our study, we aimed to explore the effects of BHD on RANKL-stimulated osteoclastogenesis *in vitro*, and glucocorticoid-induced bone loss *in vivo*, and further reveal its mechanism of action at the gene and molecular level.

## Materials and methods

### Reagents and materials

Cell counting kit-8 (cck-8) and DAPI were purchased from Beyotime (Shanghai, China). Recombinant RANKL and M-CSF were purchased from R&D (Minneapolis, MN, United States). Trypsin-EDTA (0.05%), fetal bovine serum (FBS), MEM Alpha basic, penicillin and streptomycin were purchased from Gibco (Rockville, MD, United States). Antibodies against p-p65 (#3033), ERK1/2 (#4695),P-ERK1/2 (#4370), JNK(#9252), P-JNK(#4668) were purchased from Cell Signaling Technology (Boston, MA, United States). Antibodies against NFATc1 (sc-7294), p65 (sc-8008),OPN(sc-73631),ALP (sc-365765) and Ctsk (sc-376803)were purchased from Santa Cruz Biotechnology (Dallas, CA, United States). goat anti-rabbit antibody, rabbit anti-mouse antibody, Antibodies against IκBα(bs-1287R), c-fos (bs-0469R), Sclerostin (10200R) and β-actin (bs-0061R) were purchased from Bioss (Beijing, China). Actin-Tracker Red Rhodamine-Phalloidin (YP0063S) were obtained from UE (Shanghai, China). Unless noted otherwise, other reagents were of the highest purity available and were obtained from Sigma-Aldrich (St. Louis, MO, United States).

### BHD preparation and detection of herbal ingredients

BHD is composed of Radix Rehmanniae, SemenCuscutae, Fructus Psoraleae, Eucommia ulmoides, Fructus Corni, Herba Cistanches, Fructus Lycii, Radix Angelicae Pubescentis, Radix Angelicae Sinensis, Myrrha, and Flos Carthami at a ratio of 3: 3: 3: 2: 2: 2: 2: 2: 2: 2: 1 (dry weight). All pills of herb were purchased from the Second Affiliated Hospital of Guangzhou University of Chinese Medicine. The mixture was boiled twice in distilled water at 100°C for 1 h each time. The combined filtrate was concentrated to 2 g/ml and stored at 4°C for later use. The pill was powdered and ultrasonically extracted using methanol with mass spectrometric purity for 20 min (40°C, 100w). The extract was then filtered using 0.45 μM nylon membrane. The filtrate was diluted and analyzed using ultra-high performance liquid chromatography tandem hybrid quadrupole-orbitrap mass spectrometry (UPLC-Q-Orbitrap-MS) under anion model. The analysis method was consulted with the previous study (Lu et al., 2015; Gao et al., 2016; Liu et al., 2020; Zhu et al., 2020). The chromatographic column was C18 column (2.0 mm i. d.×100 mm, 2.6 μm, Thermo Fisher Scientific Co., Waltham, MA, USA.). The mobile phase consisted of A (0.1% formic acid in water) and B (methanol) with gradient elution: 0–5 min 10% B, 5–8.5 min 100% B; 8.5–9.5 min 100% B, 9.5–9.51 min 10% B, 9.51–12 min 10%B. The flow rate was 0.3 ml/min and injection volume was 3 μl. Column temperature was 40°C. The MS were run at the following conditions: scan range 60–900 Da, ion spray voltage 4500 V, ion source heater 450°C, 30 psi curtain gas (CUR, N2), 50 psi Nebulizing gas (GS1, air), 50 psi TurboIonSpray (TIS) gas (GS2, air). The m/z values of corresponding chemical formulas of psoralen, ferulic acid, and osthole were extracted using the TraceFinder software. (Supplementary Table S1, Supplementary Figures S1–S3).

### Cell culture

Mouse macrophage cell line RAW264.7, a generous gift from the laboratory of the Guangzhou Red Cross Hospital, Guangdong Province. Cells were cultured in α-MEM supplemented with 10% of FBS, P/S (100U/ml) incubated overnight at 37°C and 5% CO2. Fresh bone marrow macrophages (BMMs) were obtained from 4 to 6 weeks old Balb/c female mice. And then grown in α-MEM medium containing 30 ng/ml M-CSF, 100U/ml P/S and 10% FBS.

### Cell counting kit-8 assay

The cell proliferation - toxicity effects of BHD on the BMMs and RAW264.7 cells were evaluated by the cck-8 assay. BMMs or RAW264.7 cells were seeded at a density of 3× 10^3^ cells/well in 96-well plates. And then, different concentrations of BHD (25, 50,100, and 200 μg/ml) were applied every 2 days for 5 or 7 days. The cck-8 assay was performed to measure the cell viability every other day by recording the absorbance at a wavelength of 450 nm on a Multiskan SkyHigh (Thermo Fisher Scientific, United States).

### *In vitro* osteoclast differentiation assay

The RAW264.7 were seeded into a 96-well plate at 5×10^3^ cells per well with culture medium containing 50 ng/ml RANKL. Similarly, BMMs (5×10^3^ cells/well) were seeded in 96-well plate using complete α-MEM containing 50 ng/ml M-CSF and 50 ng/ml RANKL. Subsequently, all cells were treated with different concentrations of BHD (50 and 200 μg/ml). The cell culture medium was replaced every 2 days until mature osteoclasts were formed. And then the cells were fixed with 4% paraformaldehyde (PFA) for 30 min. Trap staining was performed according to the manufacturer’s instructions. Trap positive multinucleated cells with three or more nuclei were considered mature osteoclasts. Visual images of osteoclasts were acquired by using an Olympus IX-73 microscope (OLYMPUS, Japan).

### Immunofluorescence staining of F-actin belts and NF-κB activity

The RAW264.7 (5×10^3^) were incubated with RANKL (50 ng/ml) and different concentrations of BHD (50 and 200 μg/ml) until observing the mature osteoclasts, cells were fixed with 4% PFA for 30 min. After fixation, permeabilized with 0.5% Triton X-100 for 10 min, and then stained with Rhodamine-Phalloidin (YP0063S, UE, China) for 20 min. And finally, DAPI to label the nuclei for 5 min. The images were obtained by using a fluorescence microscope. RAW264.7 cells were seeded on confocal dish. the cells were pretreated with concentrations of BHD (200ug/ml) for 60 min before stimulation by RANKL for 30 min. Then, cells were washed 3 times by PBS, fixed in 4% PFA for 15 min and washed 3 times by PBS again. The membrane of cells was permeated by 0.2% Triton-X100 diluted in PBS for 20 min and washed 3 times by PBS. Next, cells were blocked with 5% bovine serum albumin solution for 60 min. We removed the blocking solution and incubated cells with antibody for p65 (1:200) overnight in the dark at 4°C. Next, after washing 3 times by PBS, cells were incubated with the diluted rabbit anti-mouse IgG as corresponding secondary antibody (1:200) for 60 min in the dark at room temperature. The cells were washed 3 times by PBS in the dark, and further stained by DAPI (Beyotime, Shanghai, China) for 10 min in the dark. The intensity of p65 fluorescence signals were detected using the confocal fluorescence microscope (TCS SPE II, Leica, Germany).

### RNA extraction and real-time PCR analysis of gene expression

The RAW264.7 were seeded in a 6-well plate (2 × 10^5^cells per well) and cultured with RANKL (50 ng/ml), and varying concentrations of BHD (50 and 200 μg/ml) for 5 days. The total RNA was extracted by using nucleozol reagent (Macherey-Nagel, GER), the cDNA was synthesized from 1 µg of total RNA by the PrimeScript RT Master Mix kit (Takara Biotechnology, Otsu, Japan). Then Real-time PCR was performed using SYBR^®^ Green Premix Pro Taq HS qPCR Kit (AG, China) on an ABI QuantStudio5 (Q5) machine. The relative expression levels of the target genes were calculated and normalized to GAPDH. The primers designed in the RT-qPCRs are presented in Table 1.

**TABLE 1:** Primer pairs.

Target gene	Primer sequence (5′-3′)	
	Forward	Reverse
Ctsk	CTC​GGC​GTT​TAA​TTT​GGG​AGA	TCG​AGA​GGG​AGG​TAT​TCT​GAG​T
Nfatc1	GGA​GAG​TCC​GAG​AAT​CGA​GAT	TTG​CAG​CTA​GGA​AGT​ACG​TCT
Acp5	CAC​TCC​CAC​CCT​GAG​ATT​TGT	CCC​CAG​AGA​CAT​GAT​GAA​GTC​A
Atp6v0d2	ACC​ACG​GAC​TAT​GGC​AAC​TTC	GTA​GGT​GAG​AAA​TGT​GCT​CAG​G
GAPDH	GGA​GCG​AGA​TCC​CTC​CAA​AAT	GGC​TGT​TGT​CAT​ACT​TCT​CAT​GG

### Western blot analysis

RAW264.7 were seeded in 6-well plates at a concentration of 2×10^6^ cells/well. The cells were stimulated with 50 ng/ml RANKL on day 0, 1, 3 and 5 in the presence of BHD at 200 μg/ml. Whole cell proteins harvested after treatment using RIPA lysis buffer (Beyotime, Shanghai, China). For the short-term Western Blotting assay, RAW264.7 were seeded in 6-well plates and cultured with culture medium until they reached 90% confluence. BHD was used to pretreat the cells for 1 h followed by 0, 10, 20, 30 and 60 min of RANKL stimulation. Total cell proteins were extracted using RIPA lysis buffer containing protease and phosphatase inhibitors.10% SDS-PAGE was used to separate proteins, and the protein bands were transferred to a nitrocellulose membrane. After blocking in 5% bovine serum albumin for 1 h, the membranes were incubated with the indicated primary antibodies at 4°C overnight. Finally, membranes were incubated with secondary antibodies for 1 h, Antibody reaction was detected using the ECL system (Bio-Rad) and then exposed to X-ray film (Bio-Rad).

### Glucocorticoid-induced osteoporosis mouse model

The animal experiments were conducted according to the principles of Ethics Committee of Guangzhou University of Chinese Medicine Laboratory Animal Center for the care and use of experimental animals (Ethic No. 20220222028). Balb/c female mice weighing 20 ± 2 g, aged 8 weeks. All mice were randomly divided into three groups: sham group (n = 6), DEX group (n = 6), and DEX + BHD group (n = 6). The mice in the sham group were fed normally and were injected with 5% ethanol intramuscularly (the same amount as Dexamethasone); the mice in the other groups were injected with Dexamethasone (722G054, Solarbio, Beijing, China) (5 mg/kg, twice a week for 6 weeks) to establish the GIOP model. then an intraperitoneal injection of BHD (16 mg/kg, every 2 days for 6 weeks) was delivered for DEX + BHD group. The sham and DEX group mice were intraperitoneally injected with PBS as a vehicle control.

### Micro-CT and histomorphometry analysis

Tibia specimens were scanned and analyzed by a micro-CT scanner (SkyScan 1,172, Bruker, Germany). The scanning was carried out using following Scanning parameter: Voltage, 80 kV; source current, 100 μA; Al 0.5 mm filter; pixel size 9 μm. We manually selected the region of interest to be analyzed at 100 slices below the horizon 0.1 mm to the growth plates at the proximal tibia. The BMD, BV/TV, Tb. N and Tb. Th were measured using CT Analyzer program. Two- and three-dimensional images were generated using Data-viewer and CTvol softwares respectively. Then all tibia specimens were decalcified in 14% EDTA (BBI, Shanghai, China) at room temperature for 3 weeks. All the samples were then embedded in paraffin and sectioned into 5-µm-thick slices. Hematoxylin and eosin (H&E), trap staining and immunohistochemistry staining were performed.

### Statistical analysis

All quantitative data were analyzed using IBM SPSS Statistics 22 software. All experimental data was collected from triplicate experiments and presented as the mean ± SD and statistical significance was determined by One-way ANOVA or Student’s t test. A possibility level of *p*-value < 0.05 was considered as statistically significant.

## Results

### BHD inhibits RANKL-induced osteoclastogenesis

**FIGURE 1:**
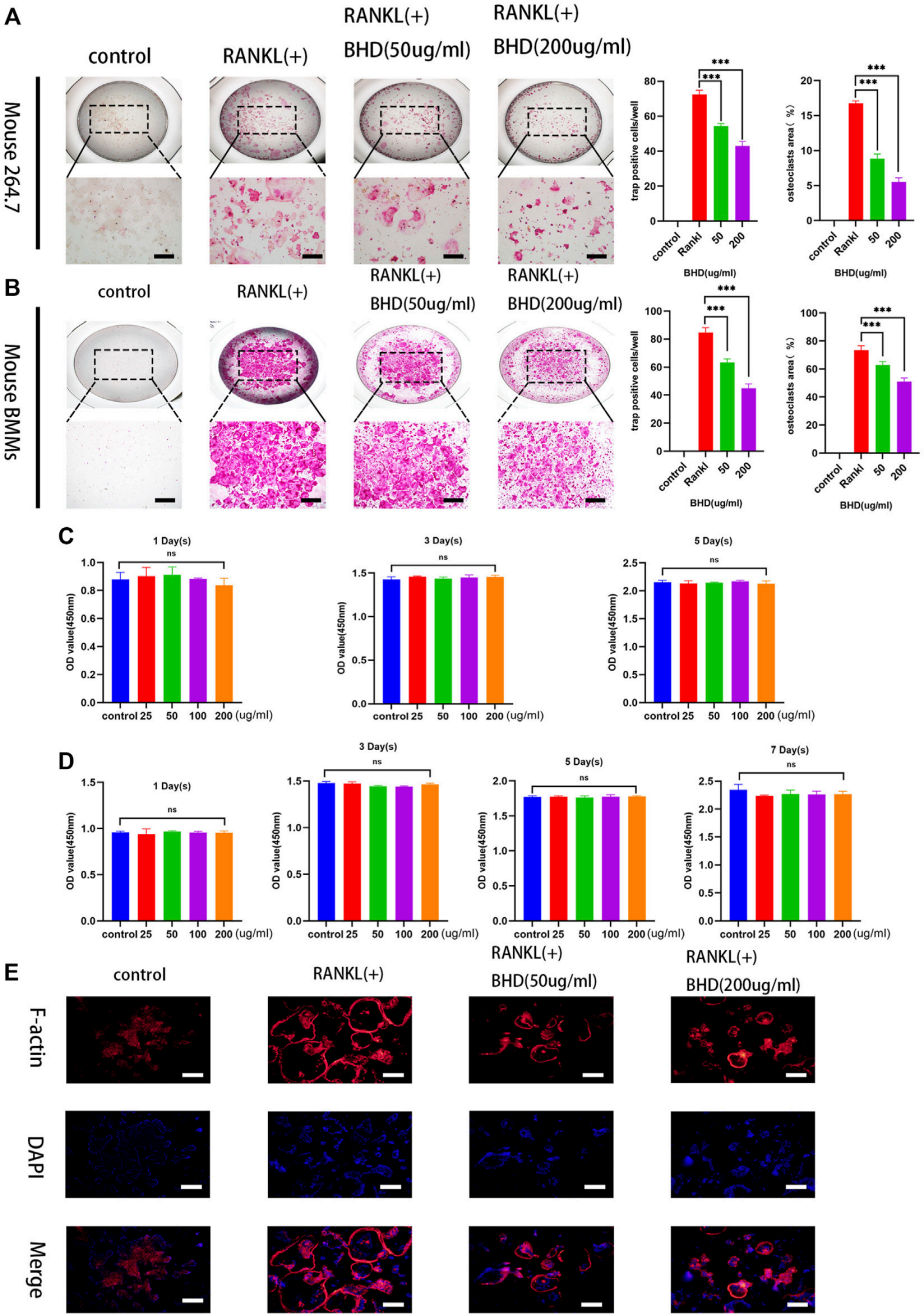
BHD suppressed osteoclastogenesis of mature osteoclasts in RAW264.7cells and BMMs **(A,B)** Mouse RAW264.7 cells (5 × 103cells) incubated with RANKL at the concentration of 50 ng/ml for 5 days with the indicated doses of BHD (50 and 200 μg/ml). Mouse BMMs (5 × 103 cells/well) incubated with RANKL and M-CSF at the concentration of 50 ng/ml for 7 days with the indicated doses of BHD (50 and 200 μg/ml). All Cells were stained using trap staining tool. Count trap-positive MNCs with 3 or more nuclei **(C,D)** The cell viability of BMMs and Raw264.7cells after pretreatment with indicated concentrations of BHD for 5 days or 7 days examined by cck-8. **(E)** The Raw264.7cells (5 × 103cells) were cultured in a-MEM containing 50 ng/ml RANKL pretreated with or without BHD for 5 days, and then the F-actin belts staining was performed. The data are shown as mean ± SD, (n = 3, three independent experiments),***p<0.001. Scalebars, 100 µm BHD: Bushen Huoxue decoction.

To explore the effect of BHD on osteoclastogenesis, two standardized osteoclast differentiation models (Raw 264.7 and BMMs) were leveraged. Results showed that BHD was able to reduce the number and size of trap-positive multinucleated osteoclasts (cells with more than three nuclei) in a dose-dependent manner (Figures 1A,B). Furthermore, to investigate whether BHD has a cytotoxic effect on BMMs and RAW264.7 cells, we performed cck-8 experiments to evaluate the viability of cells. The results showed that different concentrations of BHD had no cytotoxic effect on RAW264.7 cells within 5 days (Figure 1C), and likewise, BHD was not cytotoxic to BMMs at 7 days (Figure 1D).

### BHD inhibits F-actin belt formation

The formation of F-actin belts is critical for the function of osteoclasts, in order to investigate the effect of BHD on the formation of the F-actin belts, we used rhodamine-Phalloidin staining to observe the information on the morphological changes of the F-actin belts. The results demonstrated that BHD significantly inhibited F-actin belts formation in a dose-dependent manner (Figure 1E).

### BHD suppresses activation of ERK, JNK, and NF-κB pathways during osteoclast formation and differentiation *in vitro*

**FIGURE 2:**
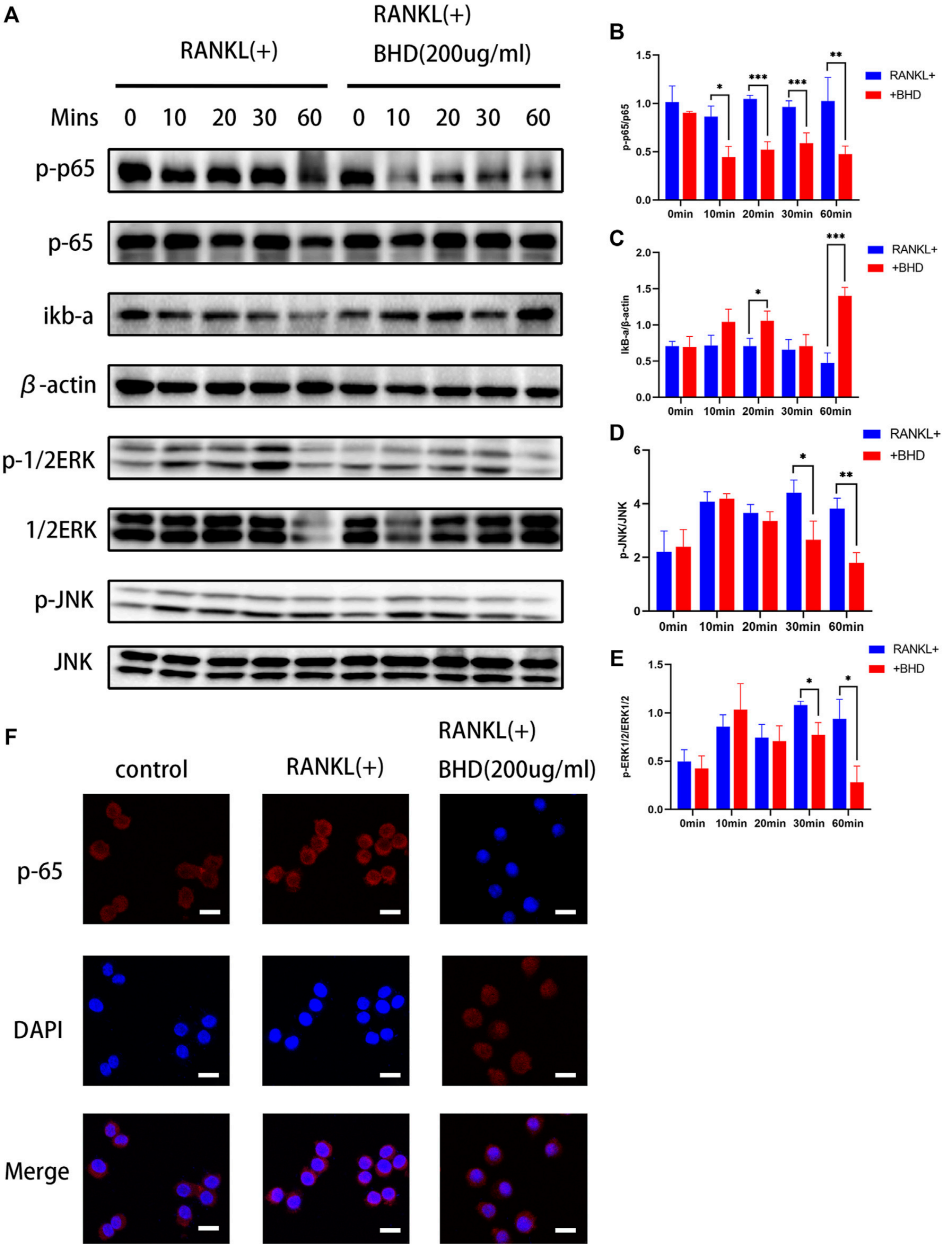
BHD suppressed RANKL-stimulated ERK, JNK and NF-κB activation **(A–E)** The protein expression levels of p-p65, p65, IκBα, p-JNK, JNK, p-ERK, ERK were examined utilizing the western blot as-says for Raw 264.7 cells pretreated with BHD at indicated concentrations. BHD inhibited RANKL-stimulated degradation of IκBα and phosphorylation of p65,ERK, and JNK. All bar charts are presented as mean ± SD, (n = 3, three independent experiments). *p<0.05,**p<0.01,***p<0.001 vs. group only treated with RANKL.**(F)** Representative immunofluorescence images of the translocation of p65. BHD suppressed RANKL-stimulated p65 translocation. Scale bars, 20 µm.

Based on the above findings, we further explored the inhibitory mechanism of BHD on osteoclastogenesis, we tested the effects of BHD on NF-κB and MAPK pathways. Firstly, in the NF-κB pathway, BHD suppressed the phosphorylation of p65 while increasing the expression of IκBα. In the MAPK signaling pathway, the phosphorylation of ERK and JNK were significantly attenuated after 30and 60 min of BHD treatment (Figures 2A–E). Meanwhile, immunofluorescence showed that BHD effectively inhibited the nuclear translocation of p65, which was consistent with the results of Western blotting (Figure 2F).

### BHD attenuates expression of osteoclast-related proteins

**FIGURE 3:**
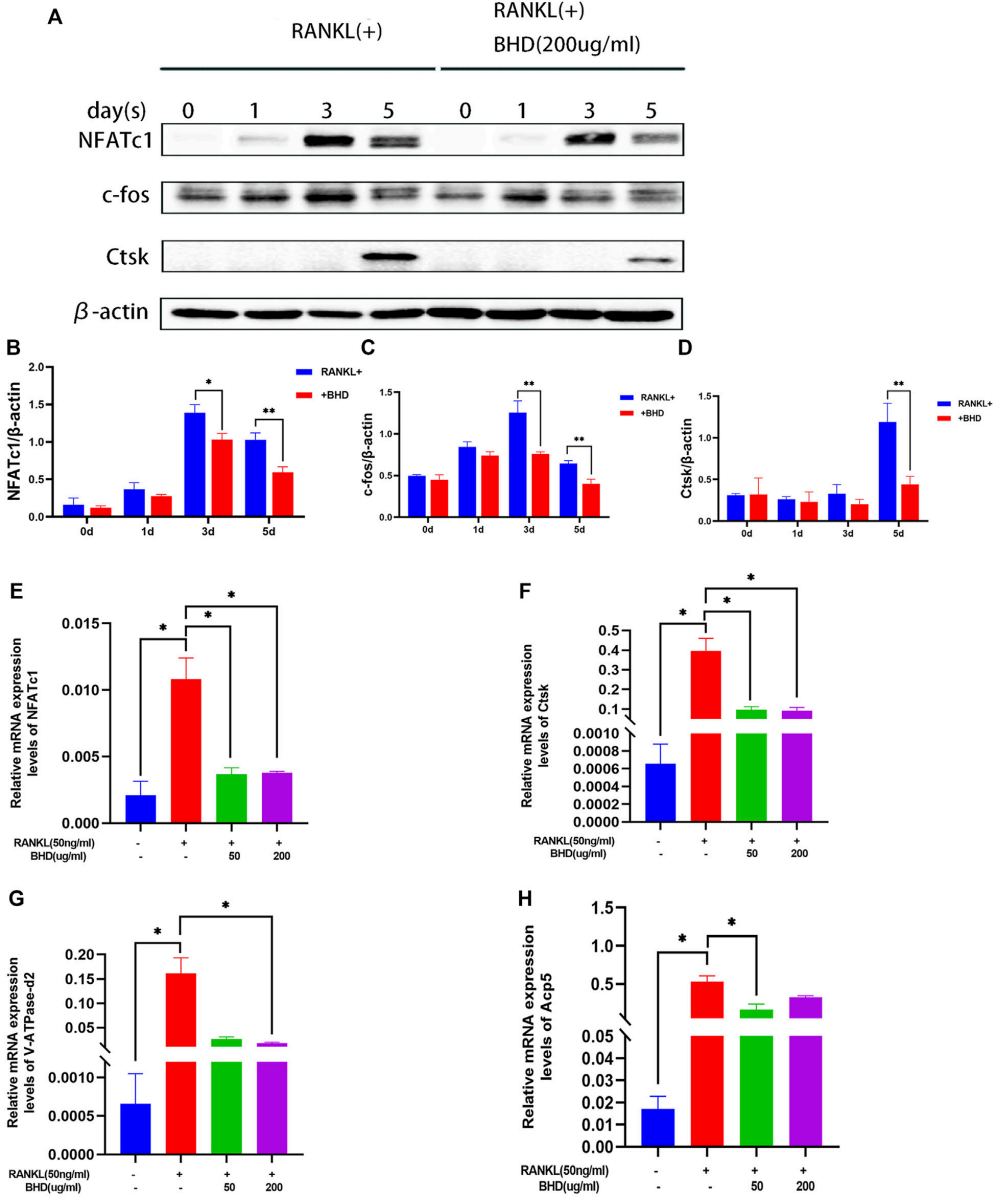
BHD suppressed RANKL-stimulated osteoclastogenesis-related protein and gene expression **(A–D)** The protein levels of NFATc1, c-fos and Ctsk of Raw 264.7cells pretreated with or without BHD for 0,1,3,5 days. BHD suppressed RANKL-stimulated protein expression levels increase in a time-dependent manner. **(E–H)** The mRNA levels of NFATc1, Ctsk, V-ATPase-d2 and Acp5 of Raw 264.7cells pretreated with or without BHD. BHD suppressed RANKL-stimulated mRNA expression levels increase of these genes. Columns in the charts were presented in a manner of mean ± SD. (n = 3, three independent experiments). *p<0.05, **p<0.01, ***p<0.001.

We then investigated whether BHD treatment inhibited key osteoclast-related proteins. NFATc1, c-fos and Ctsk are all downstream of the MAPK pathway and are key regulators in the process of RANKL-induced osteoclastogenesis. The protein expression of NFATc1, c-fos and Ctsk in RAW264.7 increased after 0, 1,3 and 5 days of RANKL stimulation, but was significantly downregulated by BHD treatment (Figures 3A–D).

### BHD represses osteoclast specific gene expressions

To determine the effect of BHD on osteoclast-specific gene expression, we performed real-time PCR assays. The expression of osteoclast-specific genes, including NFATc1, Ctsk, Acp5, V-ATPase-d2. The results showed that RANKL up-regulated the expression of these genes in mature osteoclasts. However, BHD markedly suppressed this upregulation. Therefore, these data suggest that BHD affected RNA (mRNA) expression of osteoclast-specific genes (Figures 3E–H).

### BHD protects against glucocorticoid -induced bone loss

**FIGURE 4:**
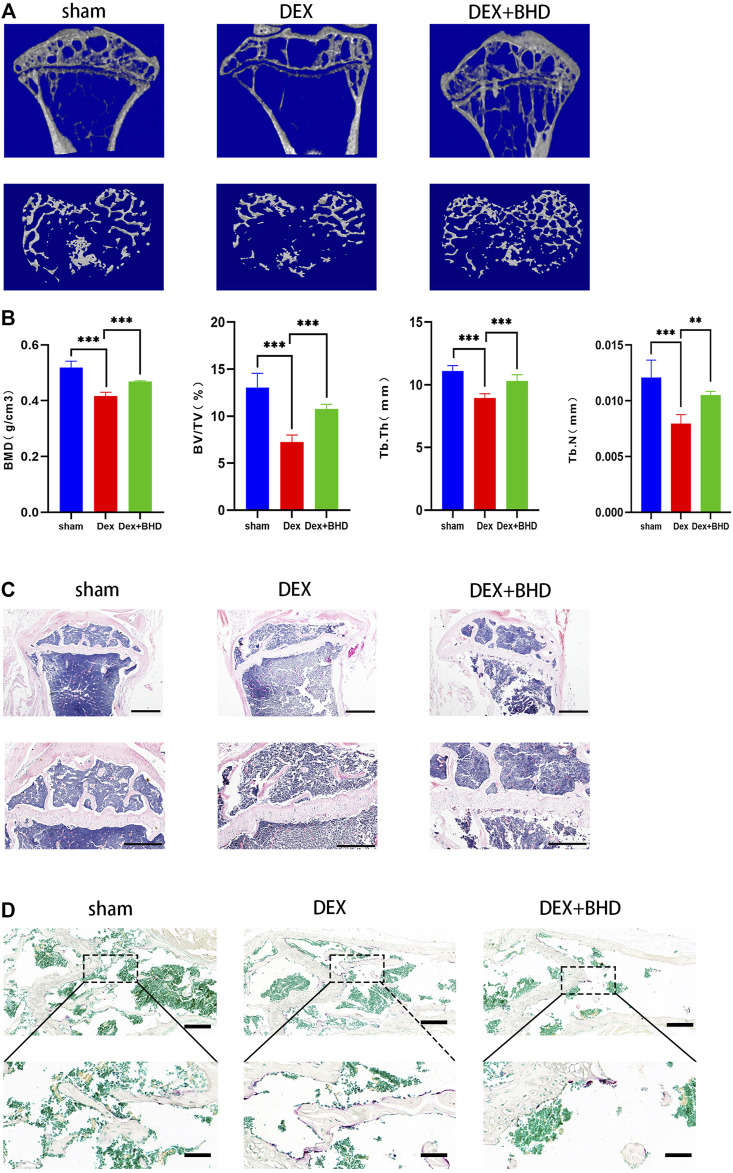
BHD prevents glucocorticoid -induced bone mass loss *in vivo*. All mice were randomly divided into three groups: sham group (n = 6), DEX group (n = 6), and DEX + BHD group (n = 6). The mice in the sham group were injected with 5% ethanol intramuscularly (the same amount as Dexamethasone); the mice in the other groups were injected with Dexamethasone (5 mg/kg, twice a week for 6 weeks) to establish the GIOP model. then an intraperitoneal injection of BHD (16 mg/kg, every 2 days for 6 weeks) was delivered for DEX + BHD group. The sham and DEX group mice were intraperitoneally injected with PBS as a vehicle control **(A)** Representative Micro-CT images of 2D and 3D demonstrating that glucocorticoid -induced bone loss was prevented by BHD treatment. **(B)** Quantitative analyses of parameters regarding to bone architecture, including BMD, BV/TV, Tb.N, Tb.Th (n = 6) **(C)** Representative images of H&E staining of decalcified bone sections, Scale bar = 200 μm or 100um. **(D)** trap staining of decalcified bone sections, Scale bar = 100 μm or 20um. All bar charts are presented as mean ± SD, *p<0.05, **p<0.01, ***p<0.001. BMD, bone mineral density; BV/TV, bone volume per tissue volume; Tb.N, trabecular number; Tb.Th, trabecular thickness.

**FIGURE 5:**
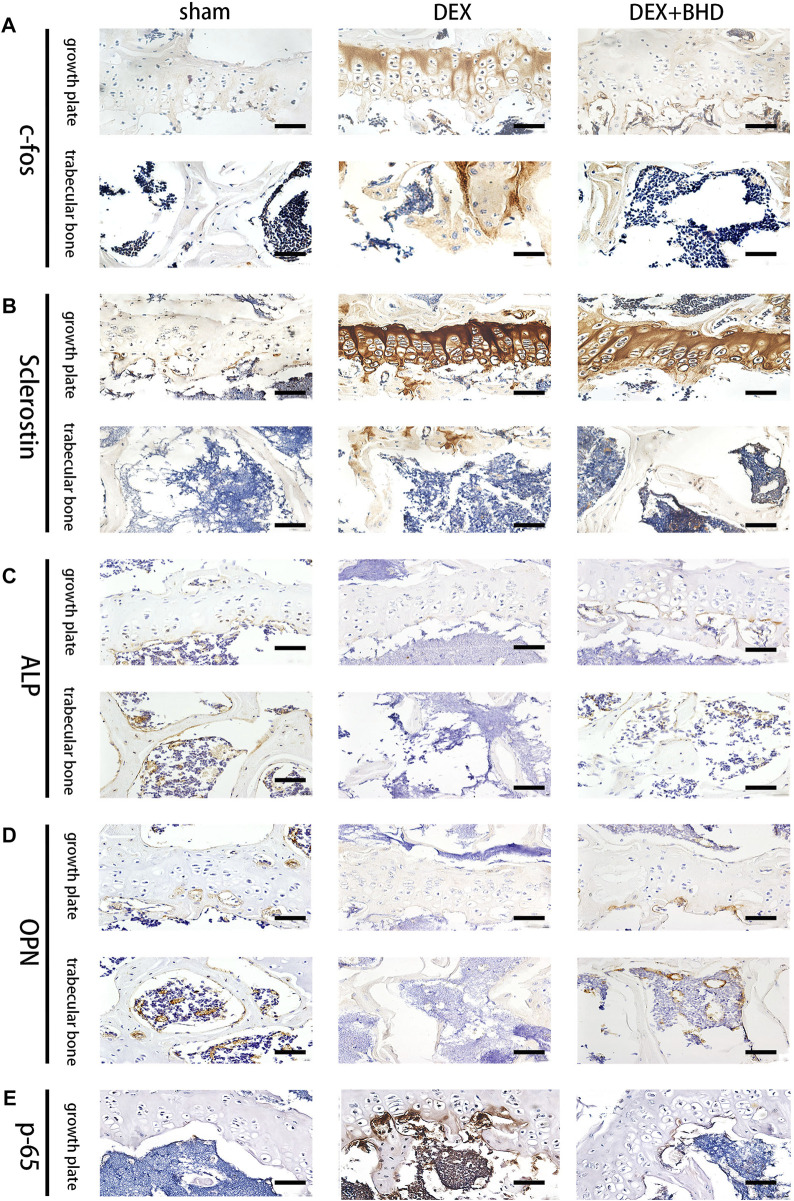
BHD affects osteoclast formation and differentiation by suppressing the expression of the NF-κB signaling pathway *in vivo*. **(A–E)** Representative images of Immunohistochemical staining of decalcified bone sections. Scale bar = 20 μm.

After establishing that BHD has the effect of attenuating osteoclastogenesis, we further explored the practical effect of BHD in preventing osteoporosis *in vivo*. Based on Micro-CT analysis, the results showed that the bone mass in the GIOP mouse model was significantly decreased compared to the control group. However, BHD prevented extensive bone loss in a mouse model of GIOP (Figure 4A). Then, the bone parameters after reconstruction were analyzed, and it was found that BMD, BV/TV, Tb. N, Tb. Th. decreased in the GIOP group, but increased after BHD treatment (Figure 4B). Likewise, H&E and TRAP staining showed that the number of osteoclasts per bone surface was reduced after BHD treatment compared with the GIOP group (Figures 4C,D). What’s more, immunohistochemical results showed a significant reduction in the expression of osteoclast-related signals c-fos and sclerostin at the growth plate and trabecular sites in the BHD treated group compared to the GIOP group, while the expression of osteogenesis-associated signals ALP and OPN was reversed (Figures 5A–D). To further investigate the mechanism, we examined the expression of p65 *in vitro*, and the results demonstrated that the expression of p65 was significantly lower in the BHD-treated group compared to the GIOP group. This result reveals that BHD attenuates bone loss in glucocorticoid-induced mice *in vitro* possibly by suppressing the expression of inflammation in the mouse osteoarthritis (Figure 5E).

## Discussion

BHD as an herbal formulation has been shown to have a variety of biological functions such as anti-inflammatory and antioxidant effects (Dong et al., 2018; Hu et al., 2020). Most importantly, BHD has proven effectiveness in the treatment of skeletal-related diseases. Recent studies reported that BHD could inhibit steroid-induced osteoclast differentiation in femoral head necrosis by promoting IGF-1 expression, stimulating osteoblast proliferation and differentiation, and extracellular matrix production. (Li et al., 2020). Besides, the active bioactive component of BHD, osteopsin, has the effect of increasing local new bone formation in a rabbit model (Wong and Rabie 2010). Our study showed that BHD inhibited RANKL-stimulated osteoclastogenesis and F-actin belts formation, but had no effect on the viability of BMMs and RAW264.7 cells. We then confirmed that the expression of osteoclastogenesis-related genes, including NFATc1, Acp5, Ctsk and V-ATPase-d2, was inhibited by attenuating the ERK, JNK and NF-κB pathways. Eventually, BHD also decreased bone mass loss through suppressing the osteoclast activity in the GIOP mouse model. These results suggest that BHD is a potential therapeutic strategy for the prevention of bone loss-related diseases.

Osteoclasts (OCs) are derived from hematopoietic progenitors of the mononuclear macrophage lineage (Boyce 2013) and are responsible for bone resorption and release of mineral matrix (Charles and Aliprantis 2014). on the contrary, osteoblasts are differentiated from mesenchymal stem cells and play an important role in bone formation (Salhotra et al., 2020). An exquisite balance between bone resorption and formation is a necessary for maintenance of bone homeostasis, any malfunction of the osteoclastic activity will break in this balance resulting in deterioration of bone structures, mass and integrity (Rachner et al., 2011; Tateiwa et al., 2019). Overactivity of osteoclasts is an important pathogenesis of GIOP, and current clinical treatments focus on anti-resorptive agents (such as denosumab and bisphosphonates) that inhibit osteoclast activity and reduce bone loss (Chotiyarnwong and McCloskey 2020). These therapies are effective, but also have long-term adverse effects, including potential breast cancer risk, thromboembolism, and osteonecrosis of the jaw (Black et al., 2012; Sakaida et al., 2017; Levin, Jiang, and Kagan 2018). Therefore, suppressing the excessive bone resorption capacity of osteoclasts should be an important therapeutic strategy. With the advantages of multi-targeting and comprehensive regulation, Chinese medicine has become an emerging area of exploration for anti-osteoporosis drugs.

Receptor activator of nuclear factor-κB ligand (RANKL) is known as the osteoclast differentiation factor (Cappariello et al., 2018). RANK is a signaling receptor for RANKL, which is mainly expressed on the surface of osteoclast precursor cells and mature osteoclasts. When RANKL is combined with RANK, the RANK signaling pathway is activated, which can maintain the activity of osteoclasts and inhibit the apoptosis of osteoclasts (Zhang et al., 2017). Our study found that BHD could inhibit RANKL-induced osteoclast formation and reduce RANKL activity in a dose-dependent manner.

The F-actin belts is an important cytoskeletal structure required for osteoclast bone resorption, formed by RANKL during terminal maturation and activation of osteoclasts. The F-actin belts is anchored to the mineralized matrix and can form a closed bone resorption cavity, which is relied on by osteoclasts to perform bone resorption function (Li et al., 2017). In the present study, we could clearly observe a large and clear F-actin belts in the RANKL-induced group, however, after BHD intervention, the size of the F-actin belts decreased with concentration. Combined with the trap results, we believe that BHD inhibits osteoclast function and maturation *in vitro*.

Based on the inhibitory effect of BHD on osteoclastogenesis *in vitro*, we further explored its mechanism. NF-κB signaling is an important downstream signaling pathway of osteoclast differentiation, RANK can cause the phosphorylation of IKKβ (inhibitor kappa B kinaseβ, IKKβ) through the combination of RANKL, resulting in the IKB (inhibitor of nuclear factor kappa-B, IKB) degradation, thus allowing NF-κB to enter the nucleus, regulating osteoclast differentiation and preventing osteoclast apoptosis (Abdelmagid et al., 2015). Other than NF-κB, increasing evidence confirmed that MAPK family members including ERKs, JNKs and p38 were also found to be closely involved in RANKL-stimulated osteoclast differentiation (Boyle, Simonet, and Lacey 2003). Among them, ERK activates the expression of c-fos, JNK phosphorylates c-fos and c- Jun (Matsushita et al., 2009). c-fos is an significant factor for NFATc1 activation and NFATc1 acts as the most peripheral transcription factor to regulating the expression of osteoclast-specific genes such as V-ATPs d2, TRAP, and cathepsin K (Chang et al., 2008; Lee et al., 2016). Our result showed that BHD treatment significantly impairs the degradation of IκBα compared to untreated group. Meanwhile, BHD also significantly attenuated RANKL-induced phosphorylation of ERK and JNK, suggesting that BHD could inhibit the activity of NF-κB, ERK, and JNK signaling pathway, thereby reducing osteoclast generation and differentiation.

Given to these *in vitro* results, we established a glucocorticoid-induced osteoporosis mouse model to further investigate whether BHD has potential treatment effect *in vivo*. The result show that BHD exhibits an obvious protective effect on glucocorticoid-induced bone loss in a mouse model which confirmed by micro-CT, trap, H&E and immunohistochemistry staining.

**FIGURE 6:**
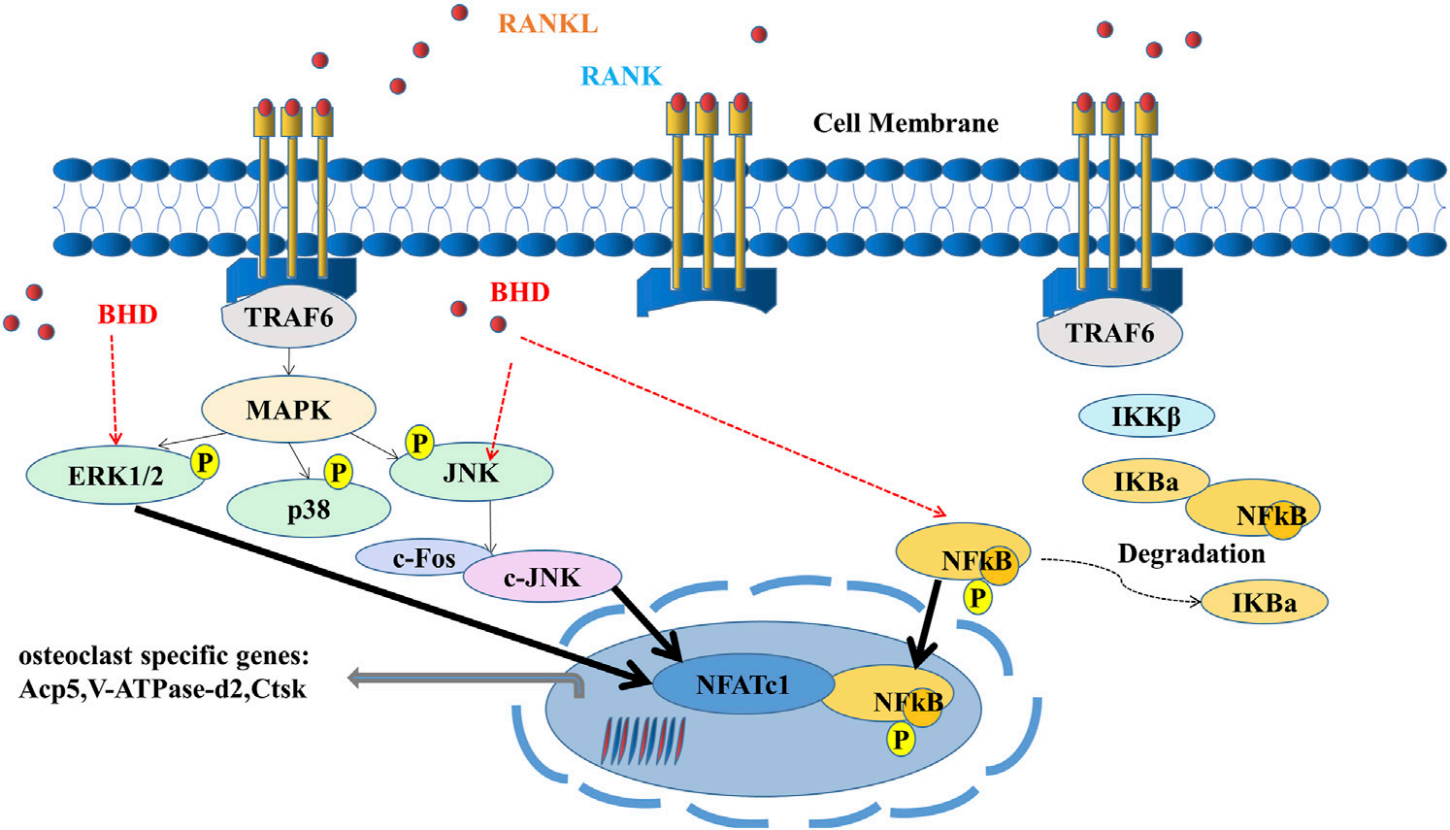
A mode of operation for the inhibition of osteoclastogenesis by BHD is proposed. ERK, JNK and NF-κB pathways are activated upon binding of RANKL to RANK. Activation of TRAF6, in turn, results in the degradation of IκBα and the upregulation of osteoclast-specific genes. Our results indicate that BHD can inhibit osteoclast formation and bone resorption by attenuating the activities of ERK, JNK, and NF-κB signaling pathways, then reduce the expression of osteoclast-related genes Acp5, V-ATPase-d2, Ctsk.

In conclusion, our study illustrated that BHD was capable to inhibit osteoclast formation and function *in vitro* and *in vivo* by suppressing ERK, JNK and NF-κB signaling (Figure 6), suggesting that BHD may prove to be a drug candidate for osteoclastogenic sicknesses like glucocorticoid-induced osteoporosis.

## Data Availability

The original contributions presented in the study are included in the article/Supplementary Material, further inquiries can be directed to the corresponding authors.
