# Corrigendum: Bushen huoxue decoction inhibits RANKL-stimulated osteoclastogenesis and glucocorticoid-induced bone loss by modulating the NF-κB, ERK, and JNK signaling pathways

**DOI:** 10.3389/fphar.2023.1148908

**Published:** 2023-02-27

**Authors:** Yamei Liu, Binlan Fu, Xiaoman Li, Chen Chen, Xican Li, Liangliang Xu, Bin Wang

**Affiliations:** ^1^ School of Basic Medical Science, Guangzhou University of Chinese Medicine, Guangzhou, China; ^2^ Laboratory of Orthopedics and Traumatology, Lingnan Medical Research Center, Guangzhou University of Chinese Medicine, Guangzhou, China; ^3^ School of Chinese Herbal Medicine, Guangzhou University of Chinese Medicine, Guangzhou, China; ^4^ The First Affiliated Hospital of Guangzhou University of Chinese Medicine, Guangzhou, China; ^5^ Department of Traumatology, The Third Affiliated Hospital of Guangzhou University of Chinese Medicine, Guangzhou, China

**Keywords:** bushen huoxue decoction, osteoporosis, NF-κB pathway, ERK pathway, JNK pathway

In the published article, there was an error in Figure 5 as published. In Figure 5E, the position of the experimental images of DEX group and DEX + BHD group is inverted, that is, the images of DEX group should appear in DEX + BHD group, while the images of DEX + BHD group should appear in DEX group. The corrected Figure 5 and its caption appear below.

**FIGURE 5:**
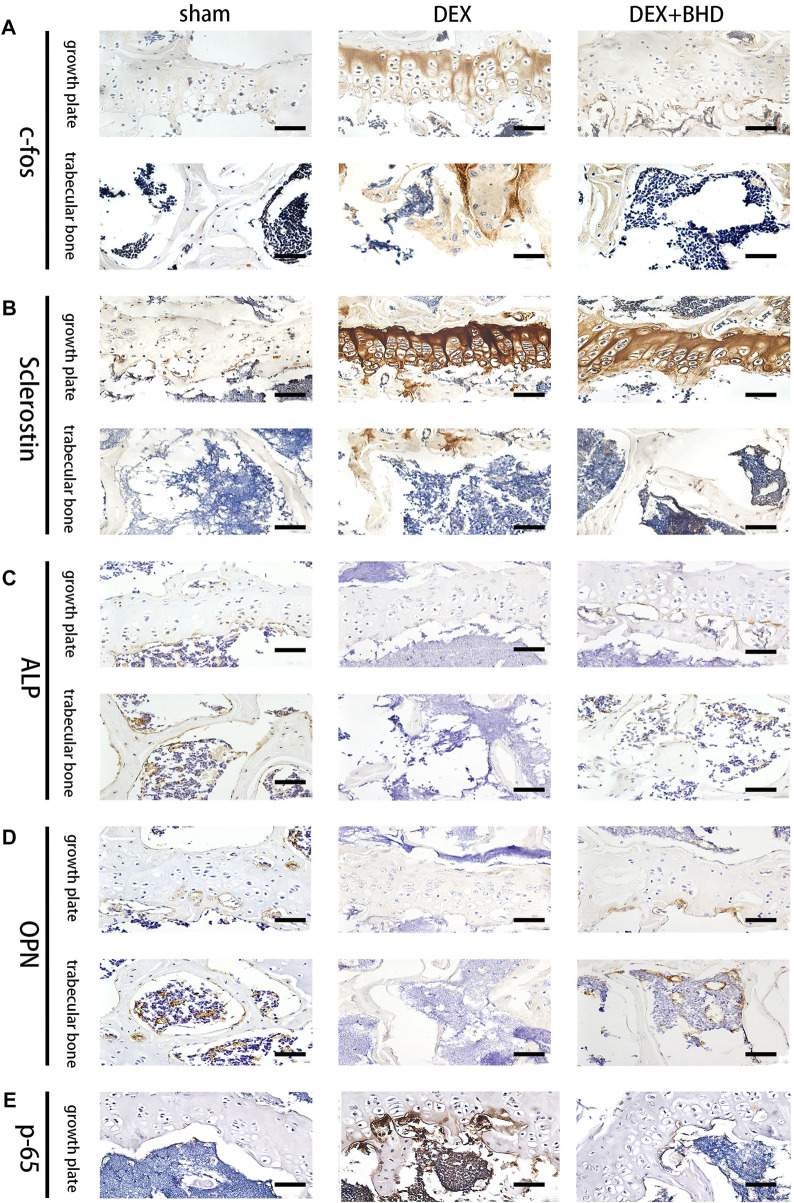
BHD affects osteoclast formation and differentiation by suppressing the expression of the NF-κB signaling pathway in vivo. **(A–E)** Representative images of Immunohistochemical staining of decalcified bone sections. Scale bar = 20 μm.

The authors apologize for this error and state that this does not change the scientific conclusions of the article in any way. The original article has been updated.

