# Supplementary figures and images for: Bushen huoxue decoction inhibits RANKL-stimulated osteoclastogenesis and glucocorticoid-induced bone loss by modulating the NF-κB, ERK, and JNK signaling pathways
Source: Front Pharmacol. 2022 Nov 18;13:1007839. doi: 10.3389/fphar.2022.1007839 (PMC9716084; doi:10.3389/fphar.2022.1007839)

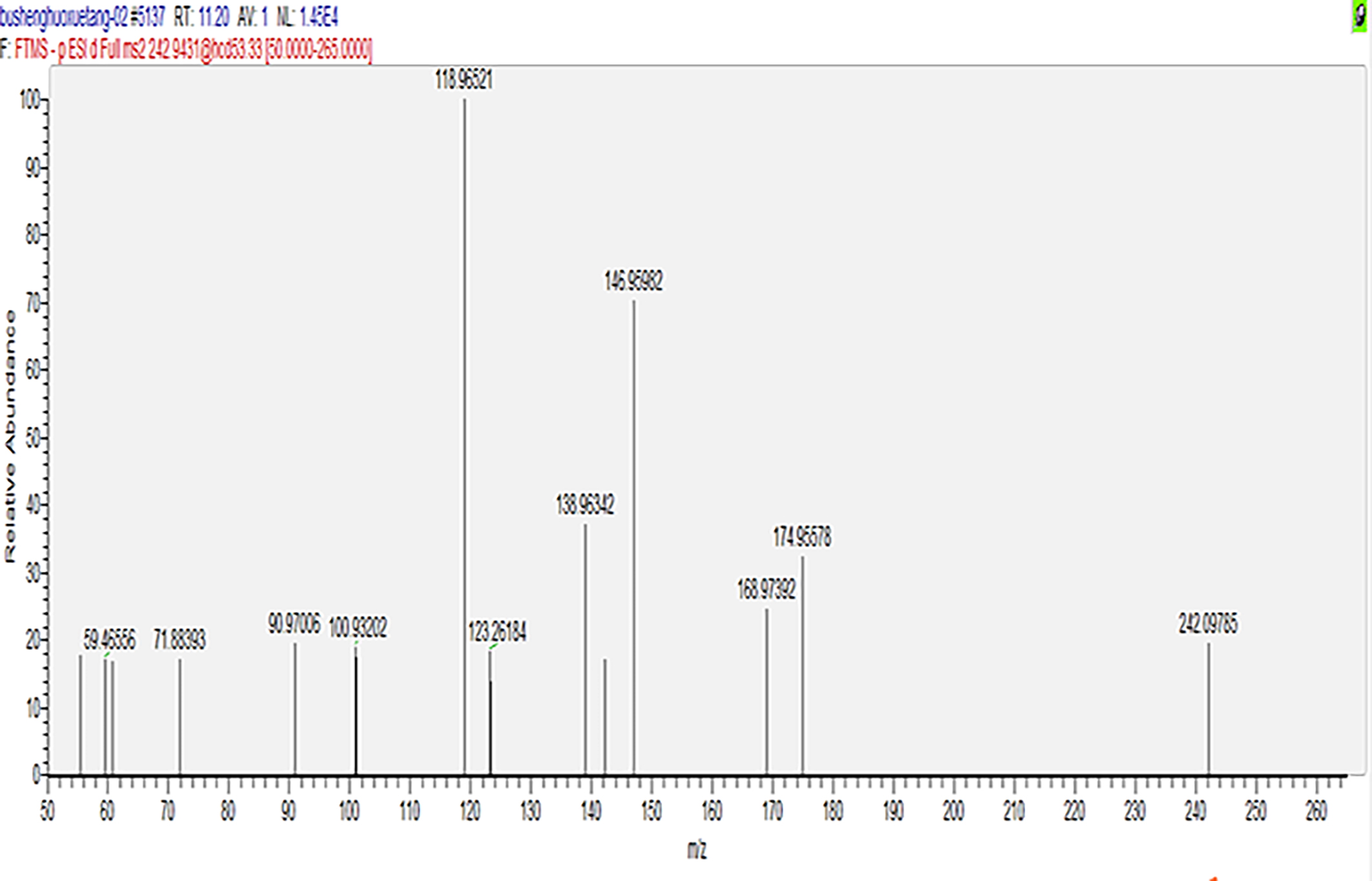

Supplement: Supplementary file 1 [file Image3.JPEG]

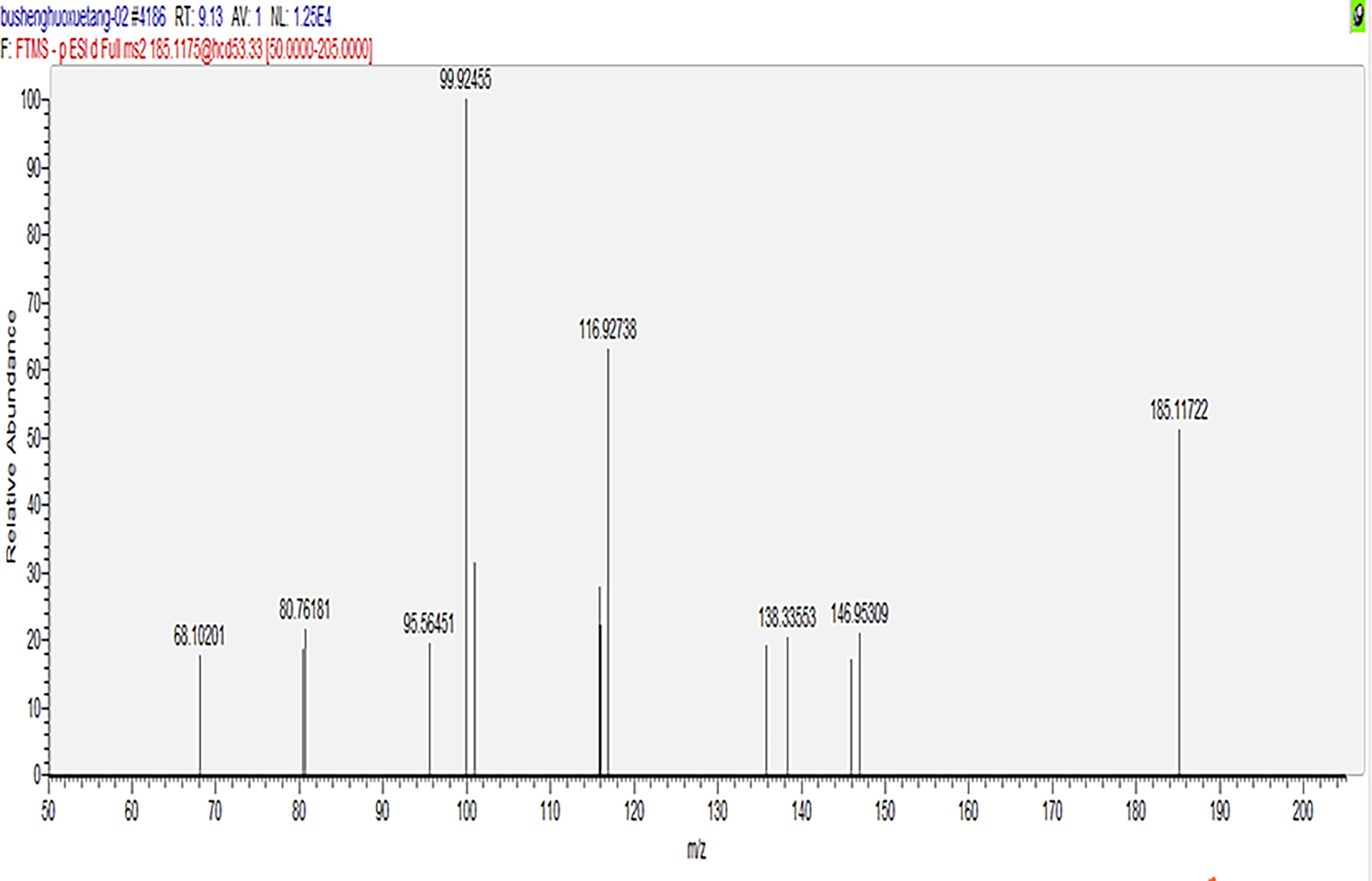

Supplement: Supplementary file 2 [file Image1.JPEG]

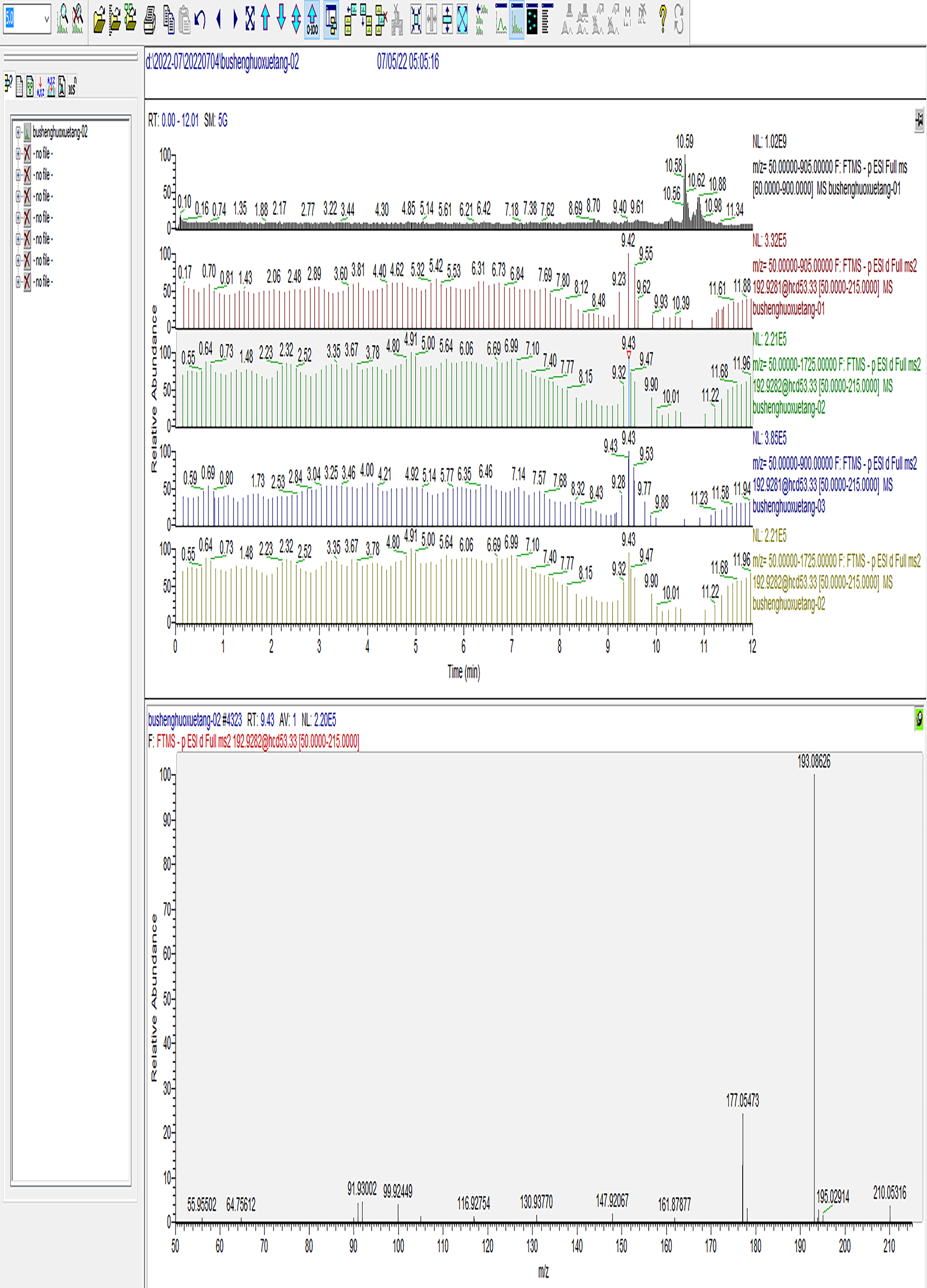

Supplement: Supplementary file 3 [file Image2.JPEG]
