# Supplementary material for: Bushen huoxue decoction inhibits RANKL-stimulated osteoclastogenesis and glucocorticoid-induced bone loss by modulating the NF-κB, ERK, and JNK signaling pathways
Source: Front Pharmacol. 2022 Nov 18;13:1007839. doi: 10.3389/fphar.2022.1007839 (PMC9716084; doi:10.3389/fphar.2022.1007839)
Supplement: Supplementary file 4 [file Table2.DOCX]

**Table S1** The retention time (min) *m/z* values of three compounds in analyzed by *UPLC-Q-Orbitrap-MS*

| Compounds name | Structure | R.T. | [M-1] | Fragments | Ref. |
| --- | --- | --- | --- | --- | --- |
| [psoralen](https://www.baidu.com/link?url=ZVFWJigBVl90yPxfBTzl7c9w7SybmfIOWNrvdxwY6t-vU58RZmW_51zx1KMNpZNtcDBdi9KV0eB_A1jDfrte3mz1-4rETgF8l0Q2mNzsHNS&wd=&eqid=a0240b83000eeac10000000662c6e1c3) |  | 9.13 | 185.11722 | 146.95309, 116.92738, 99.92455 | [1] |
| ferulic acid |  | 9.43 | 193.08626 | 177.05473, 147.92047 | [2] |
| osthole |  | 11.20 | 242.09785 | 174.95578, 168.97692, 146.95982, 118.96521 | [3] |

Note: The original UPLC-Q-Orbitrap-MS spectra were listed in S1-S3.

**References**

[1] Gao, Q.; Yan, C.; Xu, Z.; Wu, Y.; Weng, Z.; Zhao, G.; Zhang, L.; He, J.; Cai, B.; Chen, Z.; Li, W. Evaluation of the influence of salt processing on pharmacokinetics of psoralen and isopsoralen in Psoralea corylifolia L. *Biomed Chromatogr*. **2016**, 30, 528-535.

[2] Zhu, H.; Guan, J.; Zhang, H.; Chang, S.; Wang, L.; Shi, J.; Feng, B.; Gu, J. Simultaneous determination of ferulic acid, paeoniflorin, and albiflorin in rat plasma by ultra-high performance liquid chromatography with tandem mass spectrometry: Application to a pharmacokinetic study of Danggui-Shaoyao-San. *J Sep Sci*. **2020**, 43, 2053-2060.

[3] Lu, Y.; Li, N.; Deng, Y.; Zhao, L.; Guo, X.; Li, F.; Xiong, Z. Simultaneous determination of icariin, naringin and osthole in rat plasma by UPLC-MS/MS and its application for pharmacokinetic study after oral administration of Gushudan capsules. *J Chromatogr B Analyt Technol Biomed Life Sci*. **2015**, 993-994, 75-80.
