# Supplementary material for: Bushen huoxue decoction inhibits RANKL-stimulated osteoclastogenesis and glucocorticoid-induced bone loss by modulating the NF-κB, ERK, and JNK signaling pathways
Source: Front Pharmacol. 2022 Nov 18;13:1007839. doi: 10.3389/fphar.2022.1007839 (PMC9716084; doi:10.3389/fphar.2022.1007839)
Supplement: Supplementary file 5 [file Table3.DOCX]

The article and raw data for the experiments covered in the article are linked below:

https://www.jianguoyun.com/p/DQFeIHkQh6n5ChjlnNkEIAA
